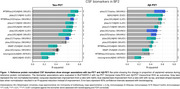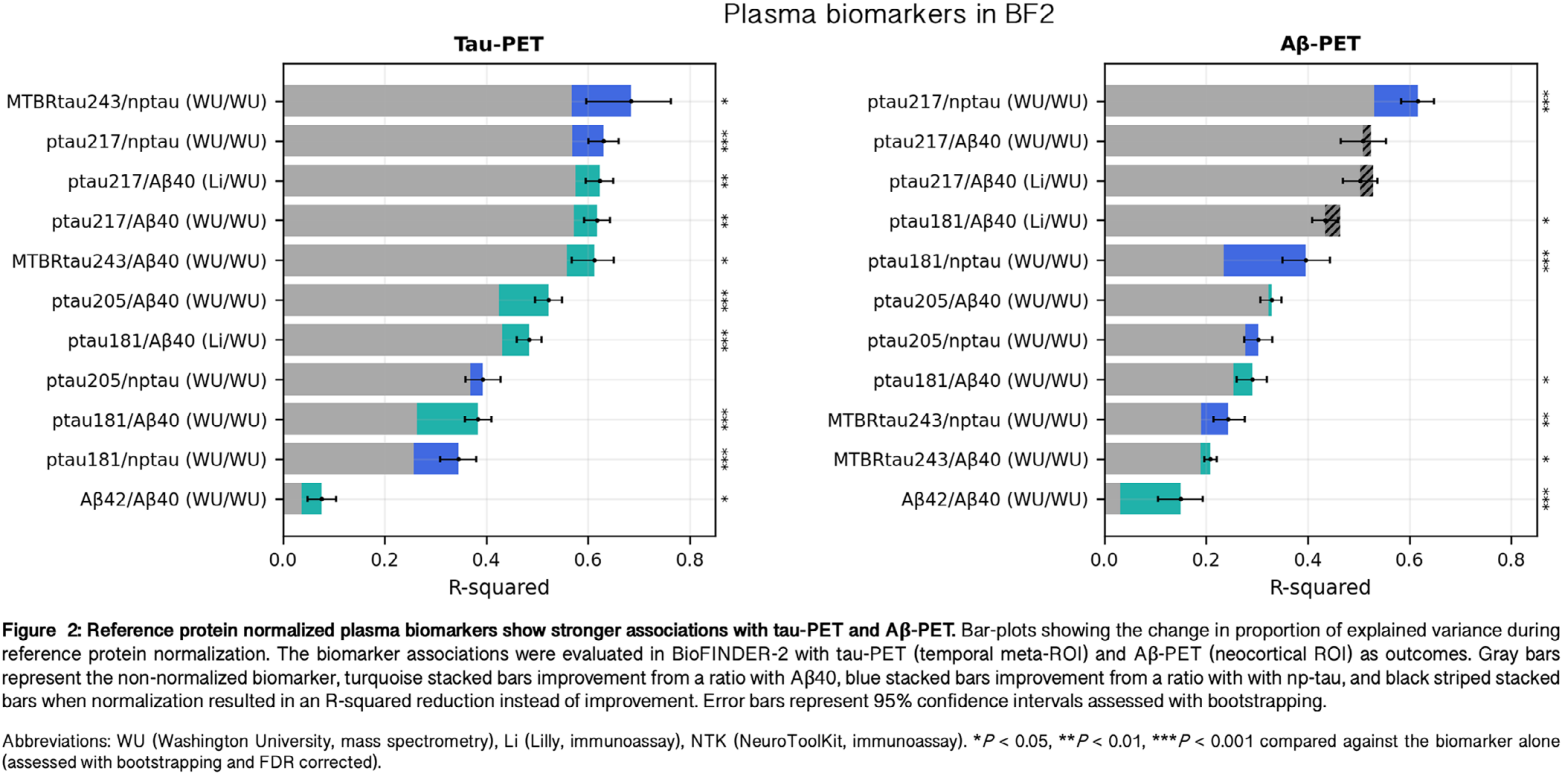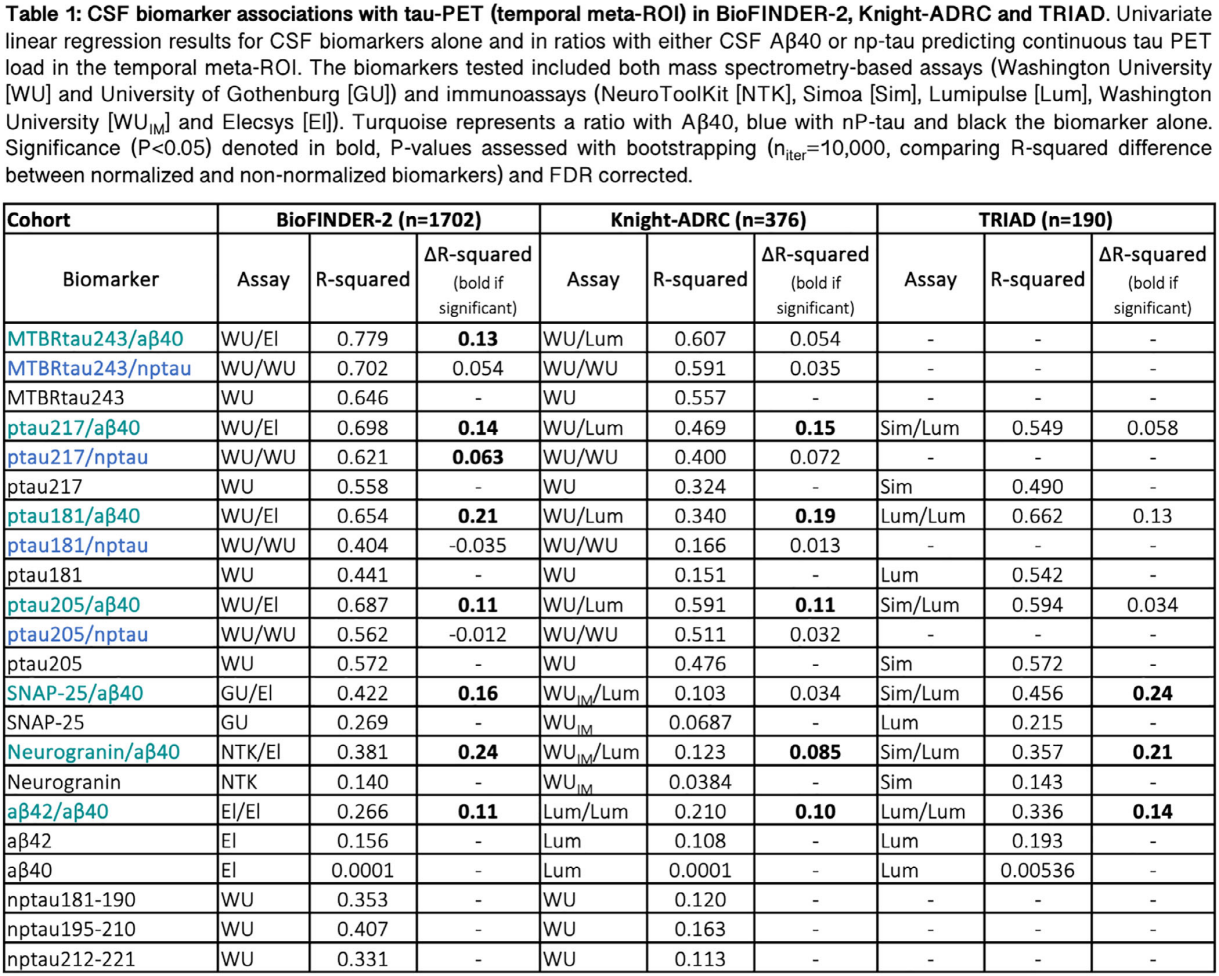# Reference proteins to improve performance of core 1 and core 2 Alzheimer's disease CSF and plasma biomarkers

**DOI:** 10.1002/alz70856_097998

**Published:** 2025-12-24

**Authors:** Linda Karlsson, Shorena Janelidze, Nicolas R. Barthélemy, Kanta Horie, Joseph Therriault, Lorenzo Gaetani, Giovanni Bellomo, Suzanne E. Schindler, Jacob W. Vogel, Ida Arvidsson, Kalle Åström, Brian A. Gordon, Cyrus A. Raji, Tammie L.S. Benzinger, John C. Morris, Johanna Nilsson, Ann Brinkmalm, Sebastian Palmqvist, Erik Stomrud, Gemma Salvadó, Alexa Pichet Binette, Massimiliano Di Filippo, Lucilla Parnetti, Pedro Rosa‐Neto, Kaj Blennow, Randall J. Bateman, Niklas Mattsson‐Carlgren, Oskar Hansson

**Affiliations:** ^1^ Clinical Memory Research Unit, Department of Clinical Sciences, Lund University, Lund, Sweden; ^2^ Department of Neurology, Washington University in St. Louis School of Medicine, St. Louis, MO, USA; ^3^ Washington University in St. Louis School of Medicine, St. Louis, MO, USA; ^4^ McGill University Research Centre for Studies in Aging, Montreal, QC, Canada; ^5^ Laboratory of Clinical Neurochemistry, Section of Neurology, University of Perugia, Perugia, Italy; ^6^ Washington University in St. Louis, St. Louis, MO, USA; ^7^ Department of Clinical Sciences Malmö, SciLifeLab, Lund University, Lund, Sweden; ^8^ Centre for Mathematical Sciences, Lund University, Lund, Sweden; ^9^ Washington University in St. Louis, School of Medicine, St. Louis, MO, USA; ^10^ Department of Psychiatry and Neurochemistry, Institute of Neuroscience and Physiology, The Sahlgrenska Academy, University of Gothenburg, Mölndal, Sweden; ^11^ Memory Clinic, Skåne University Hospital, Malmö, Skåne, Sweden; ^12^ Clinical Memory Research Unit, Department of Clinical Sciences Malmö, Lund University, Lund, Sweden; ^13^ Barcelonaβeta Brain Research Center (BBRC), Pasqual Maragall Foundation, Barcelona, Spain; ^14^ Université de Montréal, Montréal, QC, Canada; ^15^ Clinical Memory Research Unit, Department of Clinical Sciences Malmö, Faculty of Medicine, Lund University, Lund, Sweden; ^16^ Centre de Recherche de l’Institut Universitaire de Gériatrie de Montréal, Montréal, QC, Canada; ^17^ Washington University School of Medicine, St. Louis, MO, USA; ^18^ Clinical Memory Research Unit, Lund University, Malmö, Skåne, Sweden

## Abstract

**Background:**

Concentration‐based fluid biomarkers represent an informative and cost‐effective way to detect and monitor Alzheimer's disease (AD) pathology. However, non‐AD‐related inter‐individual variation in biofluids can also affect biomarker concentrations. We previously identified several reference proteins that, in the AT(N) classification framework, improved concordance between CSF Aβ42 and Aβ‐positron emission tomography (PET), as well as between CSF *p*‐tau181 and tau‐PET.^1^ However, it is still unclear what effect reference proteins have on the relationship between CSF AD biomarkers and the *load* of AD pathology. It is also unclear if *plasma* AD biomarkers can be improved by accounting for reference proteins in a similar manner.

**Methods:**

Using the Swedish BioFINDER‐2 cohort (*n* = 1702, 50.7% male, mean [SD] age 68.4 [12.2] years), we compared the associations between tau/Aβ‐PET load and CSF biomarkers (MTBR‐tau243, *p*‐tau217, *p*‐tau181, *p*‐tau205, Aβ42, SNAP‐25, neurogranin) alone versus in a ratio with a reference protein (e.g. CSF Aβ40 or non‐phosphorylated tau [np‐tau]) in univariate linear regression models. We repeated this analysis for plasma biomarkers.

**Results:**

CSF Aβ40 normalization significantly strengthened the associations of several core CSF AD biomarkers, including CSF MTBR‐tau243, *p*‐tau isoforms and synaptic biomarkers, with tau‐PET (ΔR^2^=0.064‐0.24) and Aβ‐PET (ΔR^2^=0.016‐0.28), Figure 1. CSF np‐tau normalization mainly improved concordance between CSF biomarkers and Aβ‐PET (ΔR^2^=‐0.0059‐0.19). The strongest association with tau‐PET was observed for MTBR‐tau243/Aβ40 (R‐squared=0.78, compared to 0.65 for non‐normalized MTBR‐tau243), and with Aβ‐PET for *p*‐tau217/np‐tau (R‐squared= 0.65, compared to 0.46 for non‐normalized *p*‐tau217). For core plasma AD biomarkers, including MTBR‐tau243 and *p*‐tau isoforms, associations with tau‐PET were enhanced by using plasma Aβ40 or np‐tau as references (ΔR^2^=0.0019‐0.14), while associations with Aβ‐PET mainly improved with np‐tau (ΔR^2^=0.018‐0.16), Figure 2. The findings were successfully replicated in Knight ADRC and TRIAD for improved biomarker associations with both tau‐PET (Table 1) and Aβ‐PET.

**Conclusions:**

Normalization to reference proteins (i.e., Aβ40 or np‐tau) enhances the associations between CSF and plasma biomarkers with the load of tau and Aβ pathology in the brain, making already high‐performing AD and synaptic fluid biomarkers even more precise.

**Reference**

1. Karlsson, L. *et al.* Cerebrospinal fluid reference proteins increase accuracy and interpretability of biomarkers for brain diseases. *Nat Commun* **15**, (2024).